# Crop Quality Improvement Through Genome Editing Strategy

**DOI:** 10.3389/fgeed.2021.819687

**Published:** 2022-01-31

**Authors:** Yihao Yang, Chenda Xu, Ziyan Shen, Changjie Yan

**Affiliations:** ^1^ Jiangsu Key Laboratory of Crop Genetics and Physiology/Key Laboratory of Plant Functional Genomics of the Ministry of Education/Jiangsu Key Laboratory of Crop Genomics and Molecular Breeding, Yangzhou, China; ^2^ Jiangsu Co-Innovation Center for Modern Production Technology of Grain Crops, Yangzhou, China; ^3^ Department of Crop Genetics and Breeding, Agricultural College of Yangzhou University, Yangzhou, China

**Keywords:** crop, quality, improvement, gene editing, CRISPR/Cas9

## Abstract

Good quality of crops has always been the most concerning aspect for breeders and consumers. However, crop quality is a complex trait affected by both the genetic systems and environmental factors, thus, it is difficult to improve through traditional breeding strategies. Recently, the CRISPR/Cas9 genome editing system, enabling efficiently targeted modification, has revolutionized the field of quality improvement in most crops. In this review, we briefly review the various genome editing ability of the CRISPR/Cas9 system, such as gene knockout, knock-in or replacement, base editing, prime editing, and gene expression regulation. In addition, we highlight the advances in crop quality improvement applying the CRISPR/Cas9 system in four main aspects: macronutrients, micronutrients, anti-nutritional factors and others. Finally, the potential challenges and future perspectives of genome editing in crop quality improvement is also discussed.

## 1 Introduction

Quality is the most important economic character of crops, determining products’ application value and market competitiveness. With the continuous improvement of people’s living standards, higher requirements are put forward for crop quality. Cultivating more nutritious, more delicious and healthier crop varieties is of great significance to improving people’s living standards and the sustainable development of social health. However, the pace of crop quality improvement has been relatively slow in the world due to the over-emphasis on demand for crop yield. The authors think that three main reasons are hindering the pace of crop quality improvement:1) The genetic control network of quality traits is extremely complex, and the available gene resources are still not abundant; 2) Crop quality traits are susceptible to environmental factors, such as fertilizer, climate and biological stress; 3) Traditional breeding methods (cross-breeding, mutation breeding and transgenic breeding) are time-consuming, random and introduce foreign genes.

A new generation of genome editing techniques, represented by regularly clustered interspaced short palindromic repeats/CRISPR-associated endonuclease 9 (CRISPR/Cas 9), is a revolutionary technology developed in the field of life sciences in recent years, which can produce predictable and heritable changes in specific locations of plant genomes. It includes deletion, insertion and replacement of base sequences to achieve precise improvement of plant traits. At the same time, compared with the traditional transgenic technology, the insertion site of the CRISPR/Cas9 expression vector is different from that of the gene-editing site. After the endogenous gene editing, the exogenous inserted plasmid can be removed by separating chromosomes during the generation of offspring gametes, thus eliminating the need to introduce exogenous genes. There is no transgenic controversy, and the application prospect is very broad. Providing a new turning point for the agricultural technology revolution.

At present, there are more and more cases of crop quality improvement using the CRISPR/Cas9 system. Here, we briefly review the various genome editing ability of the CRISPR/Cas9 system, summarized the recent progress in CRISPR/Cas9-mediated crop quality improvement, and further discussed potential challenges and future perspectives of genome editing in crop quality improvement.

## 2 Establishment of the CRISPR/Cas9 System

The CRISPR/Cas system is widely present in bacterial and archaebacterial genomes and is part of the adaptive immune system of microorganisms (Mojica et al., 2005; Grissa et al., 2007; Jinek et al., 2012). It consists of two core components: the clustered regularly interspaced short palindromic repeats (CRISPR) and Cas protein. The CRISPR constitutes 20–50 bp palindromic repeat sequence (Repeat), non-repeating 20–58 bp spacer sequence (Spacer) and AT-rich leading sequences (Leader). Cas protein acting as DNA endonuclease helps bacteria acquire new space sequences, essential for the bacterial immune system. According to the number and function of Cas proteins, CRISPR/Cas system has been divided into two classes and five types (I–V) (Makarova et al., 2011; Makarova and Koonin, 2015). Type I, III and IV belong to class I requiring multiple Cas proteins to form complex to work cooperatively. At the same time, type II and V belongs to class II interfering with target genes using only one single Cas protein. The immune process of the CRISPR/Cas system can be divided into three stages in bacteria (Makarova et al., 2011): 1) When foreign DNA infects bacteria, short DNA homologous fragments from protospacer sequences on bacteriophages or plasmids are integrated into the downstream of CRISPR leading sequence to form new space sequences; 2) CRISPR is induced to be transcribed into long RNA precursors (pre-crRNA), which are then truncated into short mature crRNAs, then the crRNAs precisely bind to trans-activating RNAs (tracrRNA) to fuses into tracrRNA/crRNA complexes; 3) The complexes regulate and guide Cas protein to precisely destroy the foreign DNA sequence, and produce DNA double-strand breaks (DSBs).

With a better understanding of the bacterial CRISPR/Cas immune system and its operational principle, scientists began to modify and apply this system to plant and animal genome editing (Cho et al., 2013; Cong et al., 2013; Feng et al., 2013; Mali et al., 2013; Ma et al., 2015; Yin et al., 2017). CRISPR/Cas9 system is the only class II type system reported for gene editing (Hsu et al., 2014). By artificial design, the tracrRNA/crRNA complex was simplified to a short guide RNA (sgRNA), which contains a ∼20 nt fragment complemented to a specific site of target genes and followed by a protospacer adjacent motif (PAM) in the target genes of interest. Under the guidance of sgRNA, DSBs are created by Cas9 nuclease at ∼3 bp upstream of the PAM motif and then repaired through the error-prone non-homologous end-joining (NHEJ) or the error-free homology-directed repair (HDR) pathways. The NHEJ repaired way usually results in gene knockout to lose protein function (Liu et al., 2019). Alternatively, the HDR pathway can be triggered when an exogenous DNA repair template is provided, resulting in the introduction of the repair template into a target genomic region (Chapman et al., 2012).

## 3 CRISPR/Cas9 System in Plant Functional Genomics Research

**FIGURE 1:**
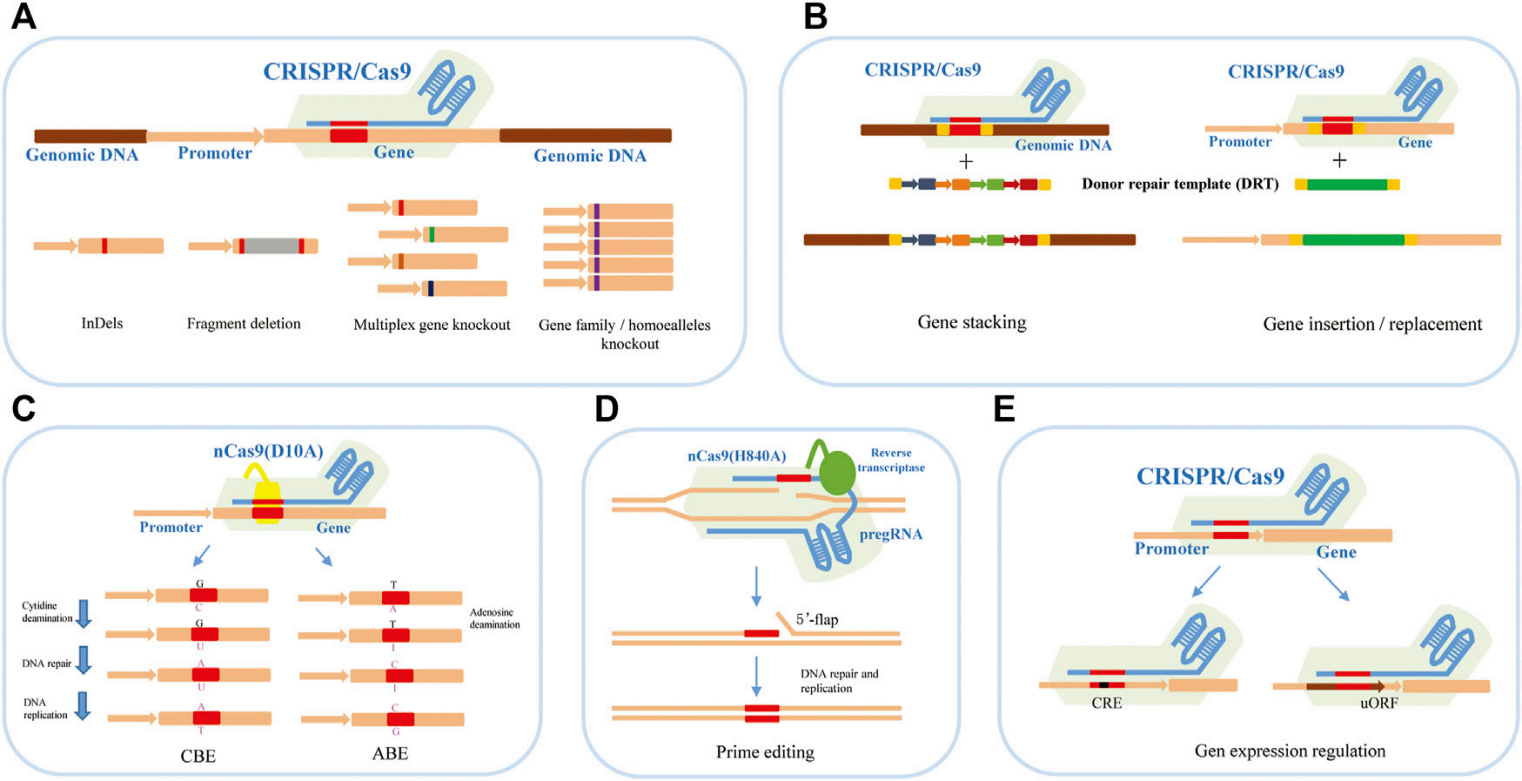
The applications of the CRISPR/Cas9 system in plant genome editing. **(A)** CRISPR/Cas9-mediated gene knockout can achieve InDels, fragment deletion, and multiplex gene knockout. **(B)** CRISPR/Cas9-mediated gene knock-in and replacement mediated by HDR can achieve gene stacking and gene insertion or replacement to produce new traits in breeding. **(C)** CRISPR/Cas9-mediated base editing for crop trait improvement including CBE-mediated C-to-T base-editing strategy and ABE-mediated A-to-G base-editing strategy. **(D)** CRISPR/Cas9-mediated prime editing for crop trait improvement. **(E)** CRISPR/Cas9-mediated gene expression regulation by editing the cis-regulatory elements and upstream open reading frames (uORFs).

At present, the applications of the CRISPR/Cas9 system in plant genome editing mainly focus on gene function research and genetic improvement of crops. It has shown various genome-editing abilities, such as gene knockout, knock-in or replacement, base editing, prime editing, and expression regulation (Figure 1).

### 3.1 Gene Knockout

Gene knockout is the most extensive application of the CRISPR/Cas9 system in plant functional genome research, which can be divided into single and multiplex gene knockout (Figure 1A). Under the guidance of sgRNA, the Cas9 nuclease cleaves the target DNA segment in the exon region of the gene to cause DSBs. The preferred NHEJ repair pathway is prone to produce insertions/deletions of one or several bases near the cutting site (Feng et al., 2014; Ma et al., 2015). When the number of inserted or missing bases is not multiple 3, the frameshift mutation will occur, and the target protein cannot be correctly encoded. It is worth noting that small insertions or deletions in edited cell lines may produce abnormal transcripts or proteins, causing unexpected effects that complicate functional analysis (Tuladhar et al., 2019). Therefore, many studies tend to produce two DSBs within a distance to delete larger genomic fragments to make true null alleles of coding and non-coding genes by NHEJ or microhomology-mediated end joining (MMEJ) repair (Owens et al., 2019; Tan et al., 2020).

Based on the high efficiency of CRISPR/Cas9 system-mediated gene knockout, multiplex gene knockout technology provided great convenience for functional analyzing the gene families, elucidating the regulation of multiple genes for complex agronomic traits, and analyzing the signal pathway in plants. Multiple sgRNAs with different targets for different genes can be designed and assembled into an expression cassette for transformation (Figure 1A). For example, our group constructed a CRISPR/Cas9 vector for targeting eight agronomic genes simultaneously based on the isocaudamer-based method. All editing genes have high mutation efficiencies in the T_0_ generation, and both heterozygous and homozygous genotypes at eight genes were obtained (Wang et al., 2015; Shen et al., 2017). In wheat, using the polycistronic tRNA strategy, Xia’s group established an efficient CRISPR-Cas9 multiplex system which can edit multiplex genes simultaneously. They succeeded in targeting mutagenesis at up to 15 genomic loci, restoring transgenic free plants and pyramiding favorable alleles in an elite wheat variety within 1 year (Luo et al., 2021). In the case of homologous genes or gene families, one sgRNA targeting the conserved sequence can also achieve good results (Li A. et al., 2018; Sánchez-León et al., 2018).

### 3.2 Gene Knock-In or Replacement

During plant domestication, some alleles are consistent and fixed within elite germplasm, such as the wheat *Q* allele (Zhang et al., 2011), rice *qSH1* (Konishi et al., 2006), maize *teosinte branched 1* (Doebley, 2004) and tomato *fw2.2* (Frary et al., 2000). Importantly, there are still many beneficial and favourable alleles in some local germplasm or related species. The replacement of endogenous genes or DNA fragments by the HDR pathway plays a useful role in crop breeding and trait improvement. HDR-mediated editing is a powerful genome-precise editing tool that enables targeted gene replacement and direct introduction of elite alleles from local or related species into commercial varieties within a few generations without any linkage drag (Figure 1B). At present, precise knock-in or replacement of gene fragments has been achieved in many plants (Svitashev et al., 2015; Luo et al., 2016; Sun et al., 2016; Begemann et al., 2017; Shi et al., 2017; Wang et al., 2017; Li J. et al., 2018; Li S. et al., 2018; Hummel et al., 2018; Li et al., 2019). However, the genes of previous reported HDR cases are often resistant genes, which rely on selection pressure, or visible makers to enrich the HDR events. The editing efficiency is very low. There are four main challenges in performing HDR in plants: 1) HDR is in constant competition with NHEJ for the repair of DSBs, but the latter is the main pathway of DSB repair (Puchta 2005; Fauser et al., 2014; Shi et al., 2017; Fan et al., 2021); 2) HDR is only active during the late S and G2 phases, while the NHEJ is active during the overall cell cycle except for mitosis (Heyer et al., 2010; Karanam et al., 2012; Truong et al., 2013; Orthwein et al., 2014); 3) The donor repair template (DRT) is limited to deliver into the nucleus/cells for HDR; 4) The timing of DSBs induction and DRT transmission in target genes is difficult to coordinate (Gil-Humanes et al., 2017; Wang et al., 2017; Li and Xia, 2020).

To make editing more efficient, various strategies have been attempted in plants. There are mainly the following aspects: 1) Increasing the amount of DRT by using the bombardment deliver method or geminivirus replicons (GVR) (Baltes et al., 2014; Svitashev et al., 2015; Sun et al., 2016; Dong et al., 2020); 2) Improving the Cas9 frequency by using the specific promotors, such as egg cell- or early embryo-specific gene promoter; 3) Coordinating the delivery of Cas protein, two single-guide RNAs (sgRNAs) and DRT by using the all-in-one vector which includes all components (Peng et al., 2019). Despite these efforts, further improving HDR frequency and delivering sufficient DRT into plant cells remains very challenging.

### 3.3 Base Editing

Single-nucleotide point (SNP) mutations are the genetic basis for sharping phenotypic diversity among individuals. Base editing refers to the transformation of a single base at a specific position in a target DNA fragment. This technology does not depend on the generation of DNA DSBs so as to avoid both the randomness of NHEJ and the low efficiency of HDR. Base editors are created by fusing an engineered base modification enzyme, such as deaminase, with the catalytically dead version of the *Streptococcus pyogenes* Cas9 (dCas9) or nickase version of Cas9 (nCas9) (D10A) and currently include cytosine base editors (CBEs) and adenine base editors (ABEs) (Figure 1C). CBEs use a naturally occurring cytidine deaminase to convert target cytosines to uracil, which catalyzes C•G to T•A conversion (Komor et al., 2016). Drawing inspiration from CBEs, adenosine deaminase would result in inosine, which is read as guanine by replication and transcription machinery. Therefore, ABEs would correct A•T to G•C mutation theoretically (Gaudelli et al., 2017). The establishment of CBEs and ABEs system enables single base editing to realize four types of base conversion and has been successfully used in crop plants (Bharat et al., 2020). Recently, some progress has been made in the study of crop gene-directed evolution using base editing technology. For example, Li et al. (2020b) developed saturated targeted endogenous mutagenesis editors (STEMES) fusing cytidine deaminase with adenosine deaminase to achieve C: G > T: A and A: T > G: C substitutions simultaneously, which facilitate directed evolution of plant genes by generating *de novo* mutations. A series of highly efficient BE toolkits were developed to achieve C-to-T mutation almost without PAM restriction, and the A-to-G mutation scope was largely expanded in rice (Zhang et al., 2021). Kuang et al. (2020) presented a base-editor-mediated gene evolution method (BEMGE), which is a powerful tool that can accurately identify functional genetic variations and develop specific traits in crop breeding programs soon.

### 3.4 Prime Editing

Although the base editing technique has been able to achieve precise C•G–T•A and A•T–G•C base conversion, it is difficult to achieve precise transversion between pyrimidine and purine bases and precise insertion or deletion of small fragments. In 2019, David R. Liu’s lab developed a revolutionary new tool for precise gene editing, the prime editor (PE), which is a search-and-replace genome-editing method and has realized all 12 kinds of base substitutions, precise insertions of up to 44 bp, deletions of up to 80 bp and combinations of these edits in human cells (Anzalone et al., 2019). PE is composed of three components: nCas9(H840A), reverse transcriptase (RT), and prime editing guide RNA (pegRNA). Compared with sgRNA of CRISPR/Cas9 system, pegRNA has an additional RNA sequence at the 3′ end, including prime binding site (PBS) that pairs with the nCas9 (H840A)-nicked ssDNA strand sequence and initiates RT, and reverse transcriptase template (RT template) that contains the genetic information for the desired mutations. Under the guidance of pegRNA, nCas9(H840A) cleaves and releases the non-target DNA strand to hybridize to the PBS in the pegRNA. Reverse transcriptase synthesizes new DNA using the RT template, and the newly synthesized DNA containing the target editing is introduced into the genome to replace the original DNA sequence through DNA repair (Figure 1D). At present, this technology has been established and optimized in plants (Lin et al., 2020). However, the editing efficiency of PE is very low and varies greatly at different loci, which cannot meet the needs of basic research and practical application (Li J. et al., 2020; Gao et al., 2021). Scientists have optimized it from different angles to improve the editing efficiency of PE in plants. For example, Lin et al. (2020) optimized prime editors for plants through codon, promoter, and editing-condition optimization. They successfully obtained the regenerated prime-edited rice plants at frequencies of up to 21.8%. Lin et al. (2021) evaluated the melting temperature of PBS and showed that 30°C leads to maximal efficiency. They also presented a dual-pegRNA approach, which uses two pegRNAs *in trans* encoding the same edits, substantially enhances PE efficiency. A web application called PlantPegDesigner was provided to simplify the design of optimal pegRNAs or pegRNA pairs.

### 3.5 Expression Regulation

Although the most frequent application of CRISPR/Cas9 is to create null alleles by targeting the coding sequences, loss-of-function mutations in coding regions may result in pleiotropic or deleterious effects (Li et al., 2020a; Hendelman et al., 2021). Numerous researches in both animals and plants have revealed that many genetic changes driving evolution, domestication, and breeding occurred in *cis*-regulatory regions, including upstream, introns, and downstream regions of genes (Wang et al., 2014; Ding et al., 2021). Compared with coding region mutations, *cis*-regulated region editing is more likely to induce small phenotypic changes by modifying the level, timing, or space of gene expression, which benefit crop improvement.

To date, the alteration of gene expression in plants genome editing has focused mainly on editing or directly deleting the *cis*-regulatory elements (CRE) in the promoter region of the target genes (Figure 1E). For example, Rodríguez-Leal et al. (2017) edited the promoters of genes that control fruit size, inflorescence branching, and plant architecture by using a multiplexed CRISPR/Cas9 promoter targeting approach. This approach obtained the artificial QTL variation and precisely regulated fruit size and other important agronomic traits in tomatoes. Hendelman et al. (2021) used a *cis*-regulatory editing system to generate a comprehensive allelic series for tomato *WUSCHEL HOMEOBOX9* (*WOX9*), which plays a species-specific role in embryo and inflorescence development. This research showed that tomato *WOX9* has a pleiotropic function, which is regulated by *cis*-sequence elements at different locations of the gene promoter region. A similar strategy, Liu et al. (2021) achieved quantitative variation of maize yield-related traits by making weak promoter alleles of CLE genes associated with increased meristem size through CRISPR-based promoter fine-tuning.

In many cases, many genes that regulate important traits require a high rate of translation to achieve crop improvement, rather than loss or reduction of function (Xu et al., 2017; Reis et al., 2020). Upstream open reading frames (uORFs) are important *cis*-regulatory elements in the 5′ leading sequence of eukaryotic mRNAs, and usually inhibit the translation initiation of downstream ORFs through ribosome stalling (Zhang et al., 2020; Kurihara, 2020). Fine-tuning the translation by regulating uORFs can effectively improve the translation efficiency of target genes for the improvement of crop traits (Figure 1E). For example, editing the uORF of *LsGGP2* increased oxidative stress tolerance and the ascorbate content of lettuce by ∼150% (Zhang et al., 2018). In *Arabidopsis thaliana*, deleting the uORF of *PHO1* increased shoot Pi content and improved shoot growth under low external Pi supply (Reis et al., 2020).

## 4 CRISPR/Cas9 System for Crop Quality Improvement

A balanced, varied, and appropriate healthy diet ensures a person’s needs for macronutrients and essential micronutrients. It protects against undernutrition, micronutrient deficiencies, and non-communicable diseases such as obesity, diabetes, heart disease, stroke, and cancer. This is the foundation of a good life. The edible part of crops is mainly composed of macronutrients (starch, protein and oil) and micronutrients (anthocyanins, vitamins, γ -aminobutyric acid *etc.*). In the past 5 years, scientists have used CRISPR/Cas9 technology to study the nutritional components of crops and flavour and storage characteristics, and achieved great success (Table 1).

**TABLE 1:** List of research on crop quality improvement by using CRISPR/Cas9 system.

Traits	Target traits	Crop	Target gene	Type of edit	References
Macronutrient improvement	Starch	Rice	*GBSSI*	Gene knockout	Ma et al. (2015)
					Zhang et al. (2017)
					Fei et al. (2019)
			*GBSSI*	Expression regulation	Huang L. et al. (2020)
					Zeng et al. (2020b)
			*GBSSI*	Base editing	Li H. et al. (2020)
					Xu Y. et al. (2021)
		Barley	*GBSSI*	Gene knockout	Zhong et al. (2018)
		Cassava	*GBSSI*	Gene knockout	Bull et al. (2018)
		Maize	*GBSSI*	Gene knockout	Gao et al., 2020;
					Dong et al. (2019)
		Rice	*SBEIIb*	Gene knockout	Sun et al. (2017)
		Wheat	*SBEIIa*	Gene knockout	Li et al. (2021)
	Proteins	Barley	*D-hordein*	Gene knockout	Li Y. et al. (2020)
		Wheat	*a-gliadin*	Gene knockout	Sánchez-León et al. (2018)
		Sorghum	*a-kafirins*	Gene knockout	Li A. et al. (2018)
		Soybean	*Gly m Bd 28 K*	Gene knockout	Sugano et al. (2020)
			*Gly m Bd 30 K*		
		Rice	*OsAAP6, OsAAP10*	Gene knockout	Wang et al. (2020)
	Oils	Rapeseed	*BnaA.FAD2.a*	Gene knockout	Okuzaki et al. (2018)
		Rapeseed	*BnaFAD2*	Gene knockout	Huang H. et al., 2020
		Rapeseed	*BnTT8*	Gene knockout	Zhai et al., 2020
		Camelina	*CsFAD2*	Gene knockout	Lee et al. (2021)
		Soybean	*FAD2-1*	Gene knockout	Al Amin et al. (2019)
		Soybean	*FAD2-2*	Gene knockout	Do et al. (2019)
		Rice	*OsFAD2-1*	Gene knockout	Abe et al. (2018)
Micronutrient improvement	Anthocyanins	Rice	*Rc*	Gene knockout	Zhu et al. (2019)
		Tomato	*ANT1*	Gene knock-in	Čermak et al. (2015)
		Tomato	*SlMYB12*	Gene knockout	Deng et al., 2018;
					Yang T. et al. (2019)
		Carrot	*DcMYB7*	Gene knockout	Xu et al. (2019)
	Vitamins	Rice	*SSU-crtI, ZmPsy*	Gene knock-in	Dong et al. (2020)
		Lettuce	*LsGGP2*	Expression regulation	Zhang et al. (2018)
	γ-aminobutyric acid	Tomato	*SlGAD2, SlGAD3*	Gene knockout	Nonaka et al. (2017)
		Rice	*GAD3*	Gene knockout	Akama et al. (2020)
		Tomato	*GABA-TP1, GABA-TP2, GABA-TP3, CAT9, SSADH.*	Gene knockout	Li R. et al. (2018)
Elimination of Anti-nutritional factors	Phytic acid	Rice	*OsPLDα1*	Gene knockout	Khan et al. (2019)
		Rapeseed	*BnITPK*	Gene knockout	Sashidhar et al. (2020)
	Heavy metals	Rice	*OsNramp5*	Gene knockout	Tang et al. (2017)
		Rice	*OsHAK1*	Gene knockout	Nieves-Cordones et al. (2017)
	Enzymatic browning	Potato	*StPPO2*	Gene knockout	González et al. (2020)
	Steroidal glycoalkaloids	Potato	*St16DOX*	Gene knockout	Nakayasu et al. (2018)
	Acrylamide	Wheat	*TaASN2*	Gene knockout	Raffan et al. (2021)
Other improvements	Fragrant aromas	Rice	*OsBADH2*	Gene knockout	Ashokkumar et al. (2020); Hui et al. (2021)
		Rice	*OsBADH2*	Gene knockout	Tang et al. (2021)
		Maize	*BADH2*	Gene knockout	Wang et al. (2021)
	Shelf-life	Banana	*MaACO1*	Gene knockout	Hu et al. (2021)
		Petunias	*PhAC 1*	Gene knockout	Xu et al. (2019)
		Tomato	*RIN*	Gene knockout	Ito et al. (2015)
		Tomato	*Cnr*	Gene knockout	Gao et al. (2019)
		Tomato	*Nor*	Gene knockout	Gao et al. (2019)
		Tomato	*ALC*	Gene replacement	Yu et al. (2017)
		Tomato	*PL*	Gene knockout	Wang D. et al. (2019)

### 4.1 Macronutrient Improvement

#### 4.1.1 Starch

Starch, a high molecular carbohydrate, is a major component of harvestable crop organs and a major energy source in the human diet and an industrial feedstock. It widely exists in maize (*Zea Mays*), rice (*Oryza sativa*), wheat (*Triticum aestivum*), barley (*Hordeum vulgare*), potato (*Solanum tuberosum*), cassava (*Manihot Esculenta*), and other starch crops. Starch consists of amylopectin and amylose and is stored as semi-crystalline granules in the chloroplasts of leaves and amyloplasts of storage organs. Amylopectin has a dendritic structure, which determines the crystallinity of starch granules. Amylose is a linear glucose polymer, accounting for only about 20% of the granules. Still, it strongly determines the physicochemical properties of starch, such as stickiness, palatability, and digestibility during cooking and processing (Jobling, 2004; Sonnewald and Kossmann, 2013; Emmambux and Taylor, 2016). At present, the genetic mechanism of starch has been well analyzed. With glucose-1-phosphoric acid (Glc-1-P) as raw material, ADPG is formed under the action of glucose pyrophosphorylase (AGPase). Amylose is synthesized under the action of grain binding starch synthase GBSSI; Amylopectin is synthesized by soluble starch synthase (SSS), branching enzyme (SBE) and debranching enzyme (DBE). At the molecular level, all the genes involved in starch synthesis and regulation can affect starch quality. Knocking out *Wx* (GBSSI) leads to the rapid reduction of amylose, amylopectin content up to nearly 100% in starch granules, referred to as waxy or glutinous starch. For example, Ma et al. (2015), Zhang et al. (2017) and Fei et al. (2019) successfully reduced the amylose content of the mutant to less than 5% by knocking out the exon region of rice waxy gene *Wx* to obtain waxy rice. The same knockout strategy is also used for barley and cassava (Bull et al., 2018; Zhong et al., 2018). Gao et al. (2020) created waxy corn hybrids by deleting large segments of a *waxy* allele using CRISPR–Cas9 in 12 elite inbred maize lines. Field trials at 25 locations showed that CRISPR-waxy hybrids were agronomically superior to introgressed hybrids. Specially, this CRISPR-Cas9 waxy corn is considered unregulated by the relevant regulations of APHIS and has been conducted a pre-commercial launch in the Midwestern United States in 2019. ‘Sweet-waxy compound corn’ is now widely favoured for its chewiness and sweetness. Dong et al. (2019) edited *SH2* and *Wx* and identified single or double mutations that can be used to produce super-sweet, waxy or sweet and waxy compound corns (SWCs) that can be used in specialty corn breeding. In addition to occurring frameshift of *Wx*, scientists are trying to fine-tune AC by manipulating *Wx* at expression, post-transcriptional, and translational levels. Rice *Wx* variants including *Wx* ^*lv*^, *Wx* ^*a*^, *Wx* ^*b*^, *Wx* ^*in*^, *Wx* ^*mw*^, *Wx* ^*op/hp*^, *Wx* ^*mp*^, *Wx* ^*mq*^, and *wx* determine the eating and cooking quality (ECQ) of rice. Huang L. et al. (2020) generated six novel *Wx* alleles by editing the region near the TATA box of the *Wx* ^*b*^ promoter, which downregulated *Wx* expression and fine-tuned grain AC. Zeng et al. (2020b) targeted the 5′UTR intronic splicing site (5′UISS) of *Wx* ^*a*^ to alter the intron-splicing efficiency and generate new quantitative trait alleles. Li et al. (2020c) and Xu Y. et al. (2021) used the CRISPR/Cas9-mediated base editing system to target regions closed to the mentioned ‘soft rice’ allele responsible sites for mild reduction of rice AC.

Foods with high amylose content and resistant starch (RS) contribute to improving human health and reducing the risk of serious noninfectious diseases, while cereal crops high in RS are not widely available (Zhu et al., 2012). Researches showed that the starch branching enzyme (*SBE*) gene controls amylopectin synthesis, and the contents of amylose and resistant starch (RS) would increase in SBE mutated crops (Shimada et al.,2006). Sun et al. (2017) conducted targeted mutagenesis of rice *SBEIIb* using CRISPR/Cas9 technology, and the AC and RS contents were significantly increased to 25.0 and 9.8%, respectively. Li et al. (2021) also conducted directed mutagenesis of *TaSBEIIa* of winter wheat and spring wheat varieties through CRISPR/Cas9, and obtained transgenic high-straight wheat with improved starch composition, structure and properties.

#### 4.1.2 Proteins

Plant seed storage proteins (SSPs) are important sources of human dietary protein, mainly from cereals and legumes. According to the solubility-linked physical properties, SSPs are classified as four fractions: water-soluble albumins, salt-soluble globulins, alcohol-soluble prolamins, and alkaline-soluble glutelins. The proportions of these four kinds of proteins in different crop seeds are different. For example, glutelin is the most abundant protein fraction in rice and comprises about 60–80% of the total SSPs, while prolamin is the dominant one in maize, wheat and barley (Yang Y. et al., 2019). Many studies have shown that the level and proportion of protein fraction content greatly impact crop quality. Manipulating seed storage protein content by CRISPR/Cas9 gene editing is an effective way to regulate seed nutritional value. In barley, D hordein is one of the storage proteins in the grain, which has a negative effect on malting quality. Li Y. et al. (2020) used CRISPR/Cas9 technology to edit the *D hordein* gene in a spring barley cultivar and obtained two mutated lines. Transcriptomic analysis and protein SDS-PAGE showed that the transcription level of *the D hordein* gene and D hordein content in the mutant was lower than that of the wild type, which provided a basis for breeding high malt quality varieties. In wheat, the α-gliadin family is the main protein group associated with celiac disease. This genetic autoimmune disorder damages the small intestine and interferes with the absorption of nutrients from food. The gliadin contains a 33-amino acid polypeptide, called 33-mer, the main immunodominant peptide in celiac patients. Sánchez-León et al. (2018) targeted a conserved region adjacent to the coding sequence for the 33-mer in the gliadin genes with two sgRNAs and obtained low gluten hexaploid and durum wheat, of which immunoreactivity was reduced by 85%. A similar knockout strategy was carried out in sorghum. A single sgRNA was designed to mutate conserved region encoding the α-kafirins endoplasmic reticulum signal peptide. Edited plants’ grain protein digestibility and lysine content significantly increased (Li A. et al., 2018). In soybean, many allergens have been identified, resulting in 5–8% of babies and 2% of adults being allergic to soybean in the United States and Europe (Heppell et al., 1987). Sugano et al. (2020) designed two sgRNAs simultaneously site-directed mutate two genes encoding the major allergens Gly m Bd 28 K and Gly m Bd 30 K in two Japanese soybean varieties and successfully obtained Cas9-free plants with no Gly m Bd 28 Kor Gly m Bd 30 K protein. In rice, grain storage protein seriously affects the quality of rice, especially the eating and cooking quality (ECQ) (Yang et al., 2015; Yang et al., 2020). Generally, the higher rice grain protein content (GPC) will lead to the worse ECQ; thus, the cultivars with good ECQ always are required to have relatively lower GPC, usually less than 7%. Wang et al. (2020) used CRISPR/Cas9 system to knock out *OsAAP6* and *OsAAP10* in three high-yielding *japonica* varieties and one *japonica* line, respectively. The protein content of the mutants was decreased, and the ECQ was increased significantly.

#### 4.1.3 Oils

Seed oils are primarily used as edible oils, and their industrial application has also been gradually increasing (Biermann et al., 2011). The most seed oil contains high content of polyunsaturated fatty acids (PUFAs) such as linoleic acid but low content of monounsaturated acids (MUFAs) such as oleic acid. Notably, PUFAs oil tends to oxidize, resulting in rancidity, off-flavours, and short shelf-life. In contrast, high MUFAs oil is 10-fold higher auto-oxidizing stable than linoleic acid, which is not only beneficial for lowering cholesterol and reducing systolic blood pressure, but also preferred for industrial uses, for example as biodiesel duels and biolubricants (O’Keefe et al., 1993; Davis et al., 2008; Terés et al., 2008). Therefore, industry and food products prefer to use high-oleic vegetable oil, and many studies have begun to alter the fatty acid composition of oilseed crops artificially. Fatty acid desaturase 2 (FAD2) catalyzes the conversion of oleic acid to linoleic acid in plants, and many studies reported that suppressing *FAD2* gene expression can develop the high-oleic oilseed crops (Okuley et al., 1994; Sivaraman et al., 2004; Jung et al., 2011; Nguyen et al., 2013; Chen et al., 2015; Lee et al., 2016; Lee et al., 2017; Wood et al., 2018). Recently, scientists have done a lot of work to improve the oil quality using CRISPR/Cas9 technology, mainly focusing on some oil crops, such as rapeseed, soybean, camelina, *etc*. Okuzaki et al. (2018) targeted the *BnaA.FAD2.a* (*FAD2_Aa*) in *B. napus* to increase the oleic acid content. Huang H. et al. (2020) designed two sgRNAs, one of which targets four copies of *BnaFAD2*. The oleic acid content in seeds of mutant increased significantly, with a maximum of more than 80% compared with wild type of 66.43%, and with a decrease in linoleic and linolenic acid content. Compared with black-seeded rape, yellow-seeded *B. napus* has the characteristics of the thinner seed coat, low lignin and polyphenol content, high oil content and high protein content, so it is widely accepted as a good-quality trait. Zhai et al. (2020) targeted *BnTT8,* which controls flavonoid accumulation in crops, to successfully obtain yellow-seeded lines with elevated oil and protein content and altered fatty acid (FA) composition using the CRISPR/Cas9 system. In hexaploid *Camelina sativa*, Lee et al. (2021) used a single guide RNA covering the common region of the three *CsFAD2* homologs*.* When all three homologous genes were knocked out, seed MUFA levels increased by nearly 80%, but with a stunted bushy phenotype. However, transformants with two pairs of *CsFAD2* homologs mutated but the other pair with wild-type heterozygous showed normal growth, and a seed MUFAs production increased up to 60%. In soybean, Al Amin et al. (2019) and Do et al. (2019) respectively mutated *FAD2-1* and *FAD2-2* loci using the CRISPR/Cas9 system to increase the oleic acid content in edited soybean plants. Besides oil crops, rice bran oil (RBO) contains many valuable healthy constituents, including oleic acid. In rice, three functional *FAD2* genes were found, and only *OsFAD2-1* is the highest expressed in rice seeds. Abe et al. (2018) disrupted the *OsFAD2-1* gene by CRISPR/Cas9 system, and the oleic acid content of homozygous knockout plants was increased to more than twice that of wild type.

### 4.2 Micronutrient Improvement

#### 4.2.1 Anthocyanins

Anthocyanins are water-soluble flavonoid compounds widely distributed in plants and confer pigmentation to plants. They are all recognized antioxidants with human health benefits, such as reducing the risk of diabetes, obesity, cardiovascular disease (CVD), and certain cancers (Wang and Stoner 2008; Tsuda 2012; Vinayagam and Xu 2015; Wallace et al., 2016). Wild rice species (*Oryza rufipogon L.*)are rich in proanthocyanidins and anthocyanidins and show red pericarp, which is regulated by two complementary genes *Rc* and *Rd. Rc* encodes a basic helix-loop-helix (bHLH) transcription factor, and *Rd* encodes a dihydroflavonol-4-reductase (DFR) protein (Sweeney et al., 2006; Furukawa et al., 2007). At present, most cultivated rice varieties produce white grain due to the frameshift mutation in the exon of the *Rc* gene. Zhu et al. (2019) successfully reverted frameshift mutation into in-frame mutations by using CRISPR/Cas9-mediated method, restoring the function of *Rc* allele and converting three elite white pericarp rice varieties into red ones with high content of proanthocyanidins and anthocyanidins. Čermák et al. (2015) inserted a 1938 bp donor template into the promoter region of tomato *ANT1* gene controlling anthocyanin biosynthesis, resulting in overexpression and ectopic accumulation of pigments in tomato tissues. In addition, consumers in different regions have different fruit colour preferences; scientists also use CRISPR/Cas9 technology to regulate genes related to anthocyanin synthesis to achieve the effect of changing fruit colour. For example, European and the American consumers prefer red fruit tomatoes, while pink fruit tomatoes are more popular in Asian countries, especially in China and Japan (Lin et al., 2014). However, most tomato breeding materials are red fruit materials. Using the CRISPR/Cas9 system, Li’s group accelerated the breeding process by disrupting the *SlMYB12* gene, a key determinant for flavonoid accumulation, of a superior red tomato inbred line to produce tomato plants with pink fruit (Deng et al., 2018; Yang T. et al., 2019). Xu et al. (2019) knocked the *DcMYB7,* which activates the expression of its DcbHLH3 partner, a structural gene in the anthocyanin biosynthetic pathway, in a solid purple carrot using CRISPR/Cas9 system resulted in carrots with yellow roots.

#### 4.2.2 Vitamins

Vitamins are a small part of the organic compounds that are needed in the human diet. We have known that vitamin deficiency is directly linked to human disease. For example, vitamin A deficiency causes visual problems, such as night blindness and even blindness in severe deficiency. However, as rice lack provitamin A (mainly β-carotene), the poor populations in the developing countries of South and Southeast Asia, where white rice is a staple food, cannot meet vitamin A intake dependency criteria. Dong et al. (2020) inserted a 5.2 Kb carotenoid biosynthesis cassette consisting of the coding sequences of *SSU-crtI* and *ZmPsy* at two genomic safe harbors in rice using CRISPR-Cas9 technology and successfully obtained marker-free rice plants with high carotenoid content in seeds. Numerous epidemiological studies have shown a positive association between dietary or plasma levels of vitamin C content and health benefits. The major source of vitamin C in the human diet is ascorbic acid (ASA) from fruit and vegetables. Zhang et al. (2018) targeted the uORF initiation codon region of *LsGGP2*, a key enzyme in vitamin C biosynthesis in lettuce, it not only increased the antioxidant stress ability of lettuce, but also increased ascorbate content by ∼150%.

#### 4.2.3 γ-aminobutyric Acid (GABA)

The γ-aminobutyric acid (GABA), a four-carbon nonprotein amino acid widely presenting in plants, functions as an inhibitory neurotransmitter in the central nervous system for animals to alleviate hypertension ((Bachtiar et al., 2015). In plants, GABA is first synthesized from its precursor glutamate by glutamate decarboxylase (GAD), and then catabolized to succinate by GABA transaminase (GABA-T) and succinic semialdehyde dehydrogenase (SSADH) in a subsequent reaction. Applying the CRISPR/Cas9 system to regulate the related genes in the GABA synthesis pathway can rapidly increase the GABA content plants and improve crops nutritional quality. Previous reports indicated that GAD has a C-terminal autoinhibitory domain that regulates enzymatic function, and deletion of this domain increases GAD activity. Nonaka et al. (2017) deleted the autoinhibitory domain of *SlGAD2* and *SlGAD3*, expressed during tomato fruit development using the CRISPR/Cas9 system, and the premature termination before the autoinhibitory domain increased GABA accumulation by 7–15 fold. Similarly, Akama et al. (2020) knocked the GAD3, which is predominantly expressed in rice seeds and obtained the edited lines with seven-fold higher levels of GABA. Li R. et al. (2018) manipulated the GABA shunt in tomatoes by targeting five key genes, namely *GABA-TP1*, *GABA-TP2*, *GABA-TP3*, *CAT9* and *SSADH.* The accumulation of GABA in the leaves and fruits of the edited lines was significantly increased, and the GABA content in the leaves of quadruple mutants was 19-fold higher than that of wild type.

### 4.3 Elimination of Anti-nutritional Factors

Anti-nutritional factors (ANFs) refer to substances in the feed that adversely affect digestion, absorption and utilization of nutrients and cause adverse physiological reactions in humans and animals, such as phytic acid (PA) and heavy metal quinones, steroidal glycoalkaloids, and free asparagine. The use of gene-editing techniques to eliminate the ANFs in crops edible parts could benefit human health.

#### 4.3.1 Phytic Acid

PA acts as a major reservoir of phosphorus in seeds from cereals to oilseeds but strongly chelates essential minerals in human and monogastric animals, leading to so-called “hidden hunger.” The lipid-dependent and lipid-independent pathways are two known phytic acid biosynthesis pathways (Bhati et al., 2014; Kuo et al., 2018). The lipid-dependent pathway involves the inositol lipid phosphatidylinositol (PI), which produces phytic acid through a continuous reaction processes. Khan et al. (2019) used the CRISPR/Cas9 system to generate mutants of a phospholipase D gene (*OsPLDα1*) to disrupt the production of phosphatidic acid and reduce the phytic acid in rice seeds. Compared with the wild type, the expression of key genes related to phytic acid biosynthesis was changed and the phytic acid content was significantly reduced in *ospldα1* mutants. In *Brassica napus. L*., the key enzyme ITPK (inositol tetrakisphosphate kinase) catalyzes the penultimate step for synthesising PA in the lipid-independent pathways (Raboy, 2009). Knocking out three functional paralogs of *BnITPK* resulted in low PA and high free phosphorus using CRISPR-Cas9 system (Sashidhar et al., 2020).

#### 4.3.2 Heavy Metals

Heavy metals, which can be taken up by crops and transported to their edible parts, is widely known to be harmful to health. Cadmium (Cd) is a highly toxic heavy metal that causes osteoporosis, kidney failure, cancer, and cardiovascular diseases for humans (Bertin and Averbeck, 2006). Rice with excessive cadmium is the main source of dietary cadmium intake. Previous researches reported that the natural resistance-associated macrophage proteins 5 (NRAMP5) mediate the root uptake of Cd (Ishikawa et al., 2012; Sasaki et al., 2012). Tang et al. (2017) designed two sequence-specific single guide RNA (sgRNA) to target exon IX of *OsNramp5* in two rice cultivars. Hydroponic culture and Cd-contaminated paddy field trials showed that Cd concentrations were dramatically decreased in shoots and roots of *osnramp5* mutants.

As a result of the Fukushima nuclear accident, massive releases of radioactive cesium (Cs) isotopes ^134^Cs (2-year half-life) and ^137^Cs (30-year half-life) are expected to have contaminated about half of Japan’s soil (Yasunari et al., 2011). Cesium (Cs) is a group I alkali metal with chemical properties similar to potassium (K). Several cloned K^+^ transporters, like HAK/KUP/KT family, can also transport Cs^+^ in the plants (Véry et al., 2014; Scherzer et al., 2015). Nieves-Cordones et al. (2017) used the CRISPR-Cas system to knock out the *OsHAK1*, resulting in a strong reduction of radioactive cesium contents in mutated plants when grown in Fukushima soil highly contaminated with ^137^Cs^+^.

#### 4.3.3 Enzymatic Browning

Enzymatic browning refers to the process in which polyphenol oxidases (PPOs) catalyze the formation of phenolic substances into quinones in the presence of oxygen, resulting in the formation of dark precipitate in fruits and vegetables and loss of nutritional quality. A lower PPO activity in plants would reduce the enzymatic browning phenotype. González et al. (2020) induced *StPPO2* gene mutations in tetraploid potato using CRISPR/Cas9 system. Compared to the control, mutations in the four alleles of the *StPPO2* gene resulted in a reduction of PPO activity by up to 69% and a 73% reduction in enzymatic browning in tubers.

#### 4.3.4 Steroidal Glycoalkaloids

Also, in potatoes, there are high levels of toxic compounds of steroidal glycoalkaloids (SGAs), α-solanine and α-chaconine, in the flowers and the tuber sprouts. Nakayasu et al. (2018) edited *St16DOX* encoding a steroid 16α-hydroxylase in SGA biosynthesis, to generate two SGA-free *St16DOX*-disrupted potato hairy root lines.

#### 4.3.5 Acrylamide

Acrylamide in food is a processing contaminant that forms from free asparagine and potentially increases the risk of developing cancer for humans. In wheat, Raffan et al. (2021) knocked out the asparagine synthetase gene *TaASN2* using four guide RNAs targeting all three homologues of *TaASN2.* Compared with the wild type, the concentration of free asparagine in seeds of the plants with all six *TaASN2* alleles edited was significantly decreased, up to 90%.

### 4.4 Other Improvements

#### 4.4.1 Fragrant Aromas

The fragrant aromas of dishes or staple food keep the mind at ease and improve appetite. The most famous example is the aromatic rice varieties basmati and jasmine rice, with a popcorn-like scent, which are popular worldwide. 2-acetyl-1-pyrroline (2AP) is the key flavour compound in rice aroma volatiles. Rice flavour is mainly controlled by recessive genes *OsBadh2/fgr* (betaine aldehyde dehydrogenase 2). It is reported that *OsBadh2* converts γ-aminobutyraldehyde (GABald) to gamma-aminobutyric acid (GABA), and the reduced or loss of BADH2 activity promotes the GABald to be converted into 2AP (Bradbury et al., 2005). Comparative sequencing revealed an 8bp deletion in the 7^th^ exon of *OsBadh2* in most fragrant rice varieties, which resulted in the loss of the original function of *Badh2*, thus producing fragrance in rice leaves and grains. Based on it, Ashokkumar et al. (2020) employed the CRISPR/Cas9 tool to target the 7^th^ exon of *OsBADH2* and created novel alleles to introduce aromas into an elite non-aromatic rice variety. Similary, Hui et al. (2021) targeted the 7^th^ exon of *OsBADH2* in no-fragrant *japonica* and *indica* varieties and provided important genetic resources for grain aroma improvement in three-line hybrid rice. Tang et al. (2021) first used CRISPR/Cas9 to delete the exon nucleotide at the exon-intron junction of *OsBADH2*, which induces the exon skipping of *OsBADH2*, resulting in high 2AP production and grain fragrance. As rice, naturally fragrant germplasm has been observed in other plants, such as soybean (Juwattanasomran et al., 2011), cucumber (Yundaeng et al., 2015), coconut (Vongvanrungruang et al., 2016), sorghum (Yundaeng et al., 2013), and mung bean (Attar et al., 2017). The 2AP accumulation all results from a loss of function, a weak allele, or lower expression of *BADH2*. However, no such germplasm was found in maize. Wang et al. (2021) generated the word’s first aromatic maize by simultaneous genome editing of the two *BADH2* genes.

#### 4.4.2 Long Shelf-Life

Crop shelf life is a key quality trait in the modern supply chain, especially for fruit and ornamental crops. The short shelf life greatly limits crops’ transportation, marketing, and storage, resulting in huge postharvest losses. Ethylene is the natural plant hormone that makes fruits ripen and flowers to senescence quickly. Therefore, genetic modification to reduce endogenous ethylene or impair the ethylene biosynthetic pathway might be an effective method to prolong the shelf life of crops (Elitzur et al., 2016). Ethylene derived from methionine is converted to S adenosylmethionine (SAM) by SAM synthase, then to 1-aminocyclopropane-1-carboxylic acid (ACC) by ACC synthase and finally to ethylene by ACC oxidase (ACO) (Yang and Hoffman, 1984). Many researches have shown that *ACO*s are involved in fruit ripening and flower senescence, and the knockout of *ACOs* can effectively increase the shelf life of crops (Do et al., 2005; Inaba et al., 2007; Huang et al.,2007). Recently, Hu et al. (2021) conducted an RNA-seq analysis on mature green bananas and identified a banana *ACO* gene *Ma07_t19730.1*. This gene can be strongly induced by ethephon and inhibited by 1-MCP to a greater extent in the pulp and peel tissues. Under the natural ripening conditions, the CRISPR/Cas9-based *MaAC O 1* (*Ma07_t19730.1*)-disrupted mutants exhibited reduced ethylene production and longer shelf life than the WT. Petunias are favoured by the floricultural industry for their different flower shapes and colours and are used as a bedding plant. However, newly produced individual flowers show rapid senescence in the mother plant. Xu et al. (2020) designed two specific sgRNAs to target *PhACO1* of petunias and successfully obtained edited lines with significantly reduced ethylene production and enhanced flower longevity. Some transcription factors (TFs) operating upstream of ethylene biosynthesis pathways also play important roles in regulating the shelf life of crops. As in other climacteric fruits, for example, tomatoes produce much ethylene during ripening. The use of naturally occurring ripening mutants increases shelf life with a delay in the ripening process, such as *Nr* (Never ripe), *alc* (alcobaca), *rin* (ripening inhibitor), *nor* (non-ripening), and *Cnr* (colorless non-ripening) (Robinson and Tomes, 1968; Tigchelaar et al., 1973; Thompson et al., 1999; Garg et al., 2008). Ito et al. (2015) knocked out the *RIN* using three sgRNAs to produce incomplete-ripening fruits in which red colour pigmentation was significantly lower than that of the wild type. Similarly, CRISPR-*Cnr* mutant lines showed delayed fruit ripening phenotype, CRISPR-*Nor* mutant lines showed partially immature fruit (Gao et al., 2019). The *alc* mutants were found to have good fruit colour, flavour and resistance to bacterial diseases (Casals et al., 2011). Using the HDR-mediated gene replacement, Yu et al. (2017) successfully replaced ^317^T of *the ALC* gene with ^317^A and created a tomato line, significantly prolonged tomato storage time and shelf life. In addition to regulating crop endogenous ethylene content, shelf life is also related to alterations in cuticle properties and remodelling of the fruit cell walls (Keegstra, 2010). Pectin, which is abundant in the primary cell wall (PCWs) and mesenchymal layer (ML) of fruits, has long been known to undergo degradation during ripening (Brummell, 2006). Uluisik et al. (2016) reported a tomato pectate lyase (*PL*) gene, which is crucial for fruit softening, and the silencing of this *PL* altered texture without affecting other aspects of ripening. Wang D. et al. (2019) used CRISPR/Cas9 technology to knock out this gene and obtained similar results.

## 5 Challenges and Perspectives

**FIGURE 2:**
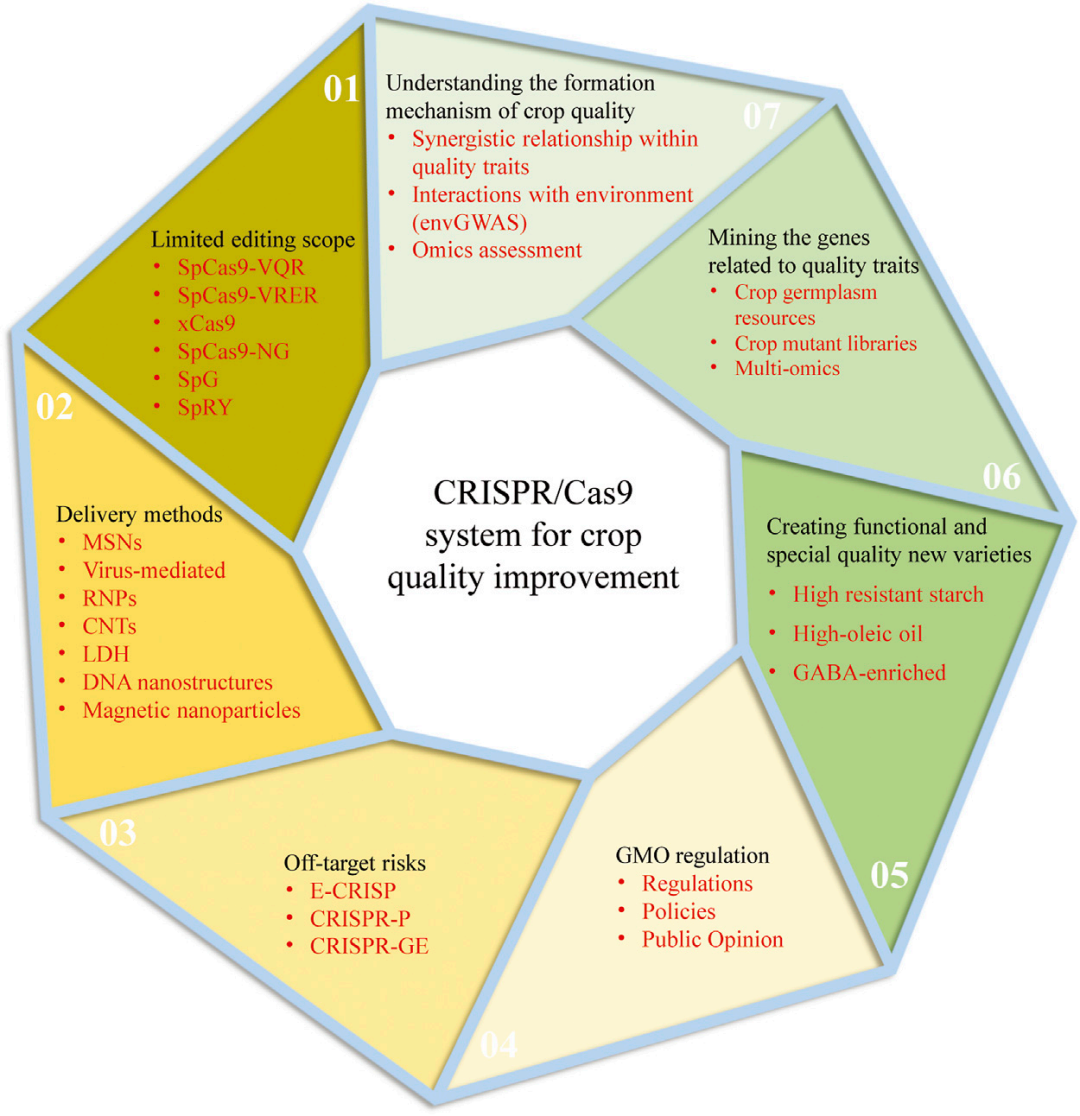
The current challenges and perspectives of the CRISPR/Cas9 system and the future development trend of crop quality improvement.

CRISPR/Cas9 system has been rapidly developed and applied since its birth in 2013 with the characteristics of simplicity, high accuracy, short cycle and low cost. However, there are still some unsolved problems in using the CRISPR/Cas9 system for genetic improvement of crop quality. The following will be analyzed from two aspects: the current challenges and perspectives of the CRISPR/Cas9 system and the future development trend of crop quality (Figure 2):

### 5.1 Challenges and Perspectives of CRISPR/Cas9 System

#### 5.1.1 Limited Editing Scope

As we all know, the targeting specificity of the CRISPR-Cas9 system is determined by two conditions: one is the specific binding of sgRNA sequence to genomic DNA sequence; another is that Cas9 protein specifically recognizes the protospacer adjacent motif (PAM) on genomic DNA. The Cas9 protein from *Streptococcus Pyogenes* (SpCas9) is the current universal Cas9 protein and specifically recognizes the NGG sequence on DNA as the PAM sequence, limiting the range of DNA sites that Cas9 protein can target. To overcome this limitation, a series of SpCas9 variants derived through protein directed evolution method has greatly expanded the editing range of the CRISPR/Cas system, Such as SpCas9-VQR, SpCas9-VRER, xCas9, SpCas9-NG, and SpG, *etc* (Hu et al., 2016; Hu et al., 2018; Wang J. et al., 2019; Zhong et al., 2019; Zeng et al., 2020a; Qin et al., 2020; Ren et al., 2021). There is hardly even a restriction on PAM sequences, such as the SpRY variant (Xu Z. et al., 2021; Ren et al., 2021). However, all the above modifications have low editing activity, and further optimization is needed to improve editing efficiency at target sites.

#### 5.1.2 Off-Target Risks

Compared with ZFNs and TALENs genome editing techniques, CRISPR/Cas9 system based on gRNA has more advantages in specific recognition. However, due to a large number of genome bases of the edited objects, similar fragments are also widely available. If these similar fragments are recognized, they will cause an off-target effect. This kind of non-specific genome editing is easy to cause uncertainty to the biological response of the edited object, which affects the reliability of this technique in research and application. With the development of high-throughput sequencing technology, many crop genome data are readily available. Based on sequence database, many software or online tools have been developed to aid in designing target sites or evaluating the outcome of genome/gene editing, such as E-CRISP (Heigwer et al., 2014), CRISPR-P (Lei et al., 2014), and CRISPR-GE (Xie et al., 2017), which will enable researchers to examine the specificity of the target sequence further and thus reduce the risk of off-target.

#### 5.1.3 Delivery Methods

The robust delivery of CRISPR-Cas9 reagent into plant cells is the basis for the effective application of CRISPR-Cas9 in plants. At present, there are two main methods for plant transformation: biological bombardment and Agrobacterium-mediated transport, but both of them have certain limitations. Agrobacterium-mediated delivery system is the most commonly used tool for plant genetic transformation, but it can only be applied to a small range of plant species or tissues due to the limitations of host genotypes; Biological bombardment can deliver biomolecules to a wide range of plant species or tissue cells, but it is inefficient and risks genome sequence destruction and tissue damage. It should be noted that these traditional methods cannot avoid the lengthy tissue culture process, and the foreign DNA fragments are needed to be integrated into the host genome, thus producing transgenic plants. Therefore, novel delivery strategies are urgently needed. The Cas9 protein-gRNA ribonucleoproteins (RNPs) is one of the most important genome-editing techniques without foreign DNA integration into plant cells. The purified Cas protein and gRNA are preassembled into a CRISPR/Cas RNP complex with complete activity *in vitro*, which is then directly introduced into plant cells through physical or chemical methods (Woo et al., 2015). This delivery method could avoid transgene integration and off-target mutations. Plant virus systems have also been modified to introduce CRISPR/Cas reagents into plant cell, which are especially helpful for homologous directed recombination mediated gene targeting (Ali et al., 2015; Gil-Humanes et al., 2017; Ellison et al., 2020). In addition, there are studies showed that nano-materials, such as Mesoporous Silica nanoparticles (MSNs), Carbon nanotubes (CNTs), layered double hydroxide (LDH) clay nanosheets, DNA nanostructures, and magnetic nanoparticles, are potential vectors for delivering various forms of CRISPR/Cas reagents (Wang P. et al., 2019; Demirer et al., 2019; Kwak et al., 2019; Zhang et al., 2019). Nano-materials can be diffused through plant cell walls without mechanical assistance and without causing tissue damage. These new genetic transformation technologies are expected to become the most important transformation methods in the future.

#### 5.1.4 GMO Regulation

At present, the safety of gene editing products is still controversial. The European Union has approved about 118 genetically modified organisms, but most of them are fed to animals, only a handful of is for human consumption directly. There is almost no genetically modified (GM) food market in Europe; gene-edited crops are considered GM products and regulated (Bruetschy, 2019). While some countries, like the United States, Canada, Australia, Japan, Argentina, and Brazil, have treated gene-edited crops (without foreign genes) as non-GMOs, which are already on the market. For example, browning resistant mushrooms created by gene editing at the University of Pennsylvania in 2016 are not regulated in the United States (Waltz, 2016). Therefore, the commercial application of gene-edited crops still needs the support and improvement of relevant regulations, policies and public opinion environment.

### 5.2 Developing Trends for Crop Quality Improvement

#### 5.2.1 Systematic Understanding Formation Mechanism of Crop Quality

Crop quality is a comprehensive and complex character manifested in the interaction with environmental factors, quality, and yield characters. Therefore, many characters often restrict each other during crop quality improvement. For example, nitrogen fertilizer as an environmental factor can promote the increase of rice yield and grain protein content. Although the yield and nutritional quality of rice were improved, the increase of grain protein content significantly decreased the rice eating and cooking quality (Yang et al., 2015; Yang et al., 2020). In the breeding process, rice yield and grain quality are often difficult to balance. In the future studies, besides focusing on a certain quality trait, attentions should also be paid to studying the synergistic relationship within different quality or yield characters and their interactions with environment. The genetic basis, molecular network and metabolic regulation mechanism of quality traits should be studied from multiple dimensions such as transcriptome, proteome and metabolome. It is worth mentioning that the envGWAS, which uses environmental or non-genetic variables as traits in GWAS to map loci associated with those variables, is an effective and popular way in such studies. This approach has been used to analysis impact of many environmental factors on crop traits, such as geographical location, climate, soil, even age and so on (Li J. et al., 2019; Millet et al., 2019; Sharma et al., 2020).

#### 5.2.2 Multi-Strategy Mining of Genes Related to Quality Traits

With the development of functional genomics and molecular biology, many genes controlling important quality traits have been successfully cloned, such as *Wx, BADH2, FAD2*, *etc.* However, compared with yield traits, the study of crop quality traits started late and was even blank in some crops with complex genetic backgrounds. Therefore, further exploration of key genes controlling quality is the precursor and basis of quality genetic improvement. We can conduct gene mining through the following strategies: 1) Crop germplasm resources are a treasure-house of abundant genetic variation. By extensively collecting crop germplasm resources, systematically evaluating quality traits and screening excellent germplasm resources, it can provide a guarantee for digging quality genes and identifying excellent alleles; 2) Using chemical, radial mutagenesis or gene-editing techniques to create crop mutant libraries to screen the mutants with changed quality traits and clone related genes; 3) Using the multi-omics method to explore quality-related genes, especially to identify key genes that respond to environmental variation, which is helpful to reveal the interaction between genetic variation and environmental variation.

#### 5.2.3 Creation of Functional and Special Quality New Varieties

At present, people are faced with the dual challenges of nutritional deficiency and overnutrition. Taking in too much sugar or lipid due to unreasonable diet structure leads to overnutrition and induces obesity, cardiovascular disease, diabetes, and kidney disease. In addition, due to unbalanced regional economic development and natural conditions, the “lack of nutrition” and “hidden hunger” problems are also more prominent, such as nutritional anaemia and vitamin A deficiency. Chronic diseases related to diet and nutrition are increasingly threatening people’s health. Based on this, the concepts of “nutrition-oriented agriculture” and “functional agriculture” have attracted more attention, and people are gradually accepting the preventive and therapeutic effects of nutrition-healthy food and functional food. At present, biofortification crops with the significant increase of one or more nutrients can be obtained by molecular design breeding or gene-editing methods, such as high resistant starch rice, giant embryo rice, golden rice, *etc*., but the progress is still slow. In the future, we need to further search for functional germplasm resources, analyze the synthesis and metabolic pathways of relevant bioactive compounds and their regulatory mechanisms, and create more new crop germplasm with high nutrition or special functions.
